# An epigenomic shift in amygdala marks the transition to maternal behaviors in alloparenting virgin female mice

**DOI:** 10.1371/journal.pone.0263632

**Published:** 2022-02-22

**Authors:** Christopher H. Seward, Michael C. Saul, Joseph M. Troy, Payam Dibaeinia, Huimin Zhang, Saurabh Sinha, Lisa J. Stubbs

**Affiliations:** 1 Pacific Northwest Research Institute, Seattle, WA, United States of America; 2 Carl R. Woese Institute for Genomic Biology, Urbana, IL, United States of America; 3 Department of Computer Science, University of Illinois at Urbana-Champaign, Urbana, IL, United States of America; Technion Israel Institute of Technology, ISRAEL

## Abstract

Adults of many species will care for young offspring that are not their own, a phenomenon called alloparenting. However, in many cases, nonparental adults must be sensitized by repeated or extended exposures to newborns before they will robustly display parental-like behaviors. To capture neurogenomic events underlying the transition to active parental caring behaviors, we analyzed brain gene expression and chromatin profiles of virgin female mice co-housed with pregnant dams during pregnancy and after birth. After an initial display of antagonistic behaviors and a surge of defense-related gene expression, we observed a dramatic shift in the chromatin landscape specifically in amygdala of the pup-exposed virgin females compared to females co-housed with mother before birth, accompanied by a dampening of anxiety-related gene expression. This epigenetic shift coincided with hypothalamic expression of the oxytocin gene and the emergence of behaviors and gene expression patterns classically associated with maternal care. The results outline a neurogenomic program associated with dramatic behavioral changes and suggest molecular networks relevant to human postpartum mental health.

## Introduction

Interactions between newborn animals and their parents are profoundly important, being critical to the well-being of the offspring and intensely consequential to the parents as well. In most mammals, parental care is typically relegated to the female that bears the offspring, with hormonal shifts that occur during pregnancy and the early postpartum period priming her for this experience. These dramatic hormonal shifts also alter a mother’s morphology, physiology, and brain structure in ways that persist far beyond the initial parenting experience [1, 2]. In addition to these physical changes [3], mothering also alters a female’s behavior, in both the immediate and the longer-term. In particular, the sight, sounds, and odors of newborns–which may otherwise be perceived as aversive by adults–become intensely rewarding and motivating to the mother [4–6]. As with other changes associated with parenting, the shift from aversion to intense affiliation and reward is coordinated by steroid hormones and a rapid surge in neuropeptide secretion around the time of birth [3]. Most significantly, a surge of oxytocin, stored during pregnancy within the paraventricular and supraoptic nuclei of the hypothalamus [7], is released to target neurons within a brain circuit central to fear, aversion, reward, and the evaluation of emotional salience [8].

New mothers are not the only individuals that can experience this switch to pup-affiliative behaviors. For example, although virgin female rats display a clearly aversive response to pup stimuli, this response can be overcome by the process of sensitization, which involves a series of repeated interactions; after sensitization, virgin rats will display robust maternal behaviors with pups [6]. In contrast, adult virgin female mice will spontaneously display certain maternal-like behaviors shortly after given first access to young pups [9, 10]. Sensitization enhances this response; virgin female mice repeatedly exposed to pups significantly increases both the range and intensity of maternal behaviors [11]. Intriguingly, it has recently been shown virgin female mice continuously co-housed with new mothers will display maternal behaviors more rapidly under the instruction and encouragement of the mothers, a process that depends upon the activation of oxytocin neurons [12]. Like mothering itself, this experience of caring for young that are not one’s own, or alloparenting, impacts future behavior. For example, juvenile female rats that have had the experience of “babysitting” younger siblings are highly motivated to display maternal behaviors in future encounters with pups [13], and sensitized adult virgin female mice demonstrate enhanced parenting skills when they have their offspring of their own [11, 14, 15]. Indeed, many of the mechanisms that reshape a mother’s brain and behavior also appear to operate in alloparenting females, where intriguingly, they are activated without the hormonal priming stimulated by pregnancy, parturition, and nursing.

Here, we investigated the functional genomics profile of the brains of co-housed virgin female mice as they transitioned from pup-naïve to a robust display of alloparenting behaviors. To identify the genomic program modulated during this transition, we examined alterations in gene expression in hypothalamus, frontal cortex, amygdala and striatum over several days of continuous pup exposure, comparing virgins co-housed with pregnant dams just before birth to those co-housed with mother and pups for one or three postnatal days. Because histone modifications have been implicated as central to the behavior of both new mothers and sensitized virgins [16], we also investigated chromatin profiles using H3K27Ac (histone H3 acetylated at lysine 27), a marker of open chromatin, in the same brain regions. The data revealed defense-related neurogenomic pathways that are silenced, and others that are activated, across the brains of co-housed alloparenting virgins as they transition to maternal behaviors and confirm an active role for histone modifications in this behavioral switch, especially within the amygdala.

## Results

### Antagonistic behavior, followed by active nurturance in virgins co-housed with mothers and pups

**Fig 1:**
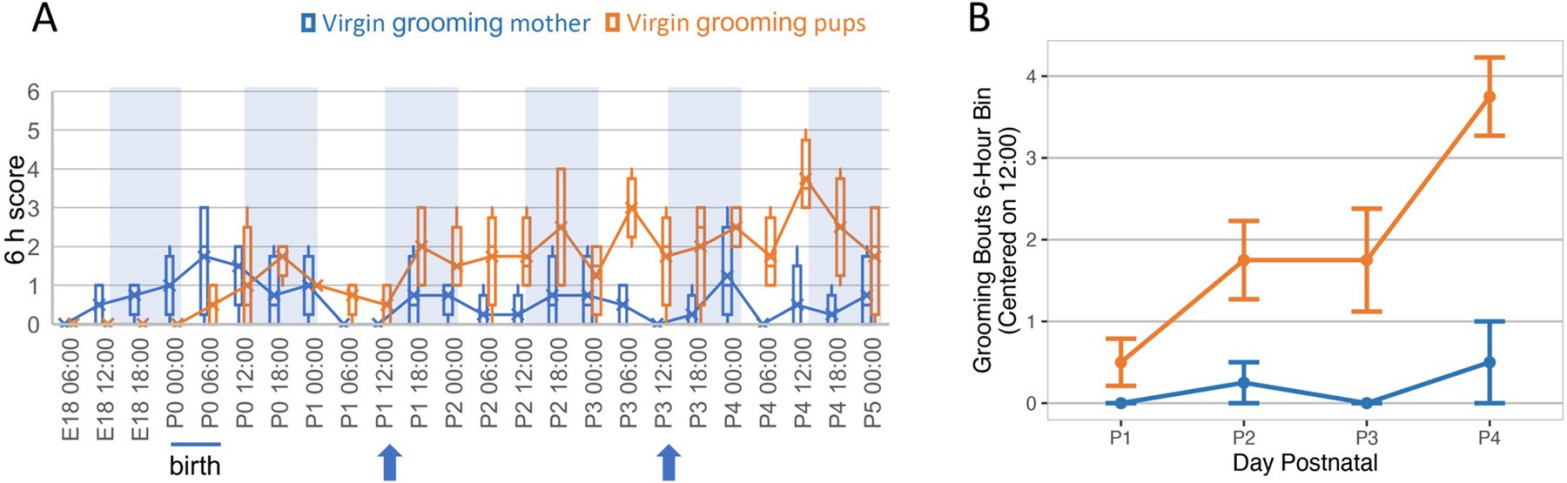
Behaviors exhibited by virgin females co-housed with new mothers and pups over six days beginning the day before birth. **(A)** Four cages of co-housed mothers and virgin females were recorded over a period of several days before and after birth and grooming behaviors (virgins to pups, plotted in orange; or virgins to mother, blue) were scored (0 or 1) in 5-minute intervals at the beginning of each hour from 12:00 am (0:00) the day before the birth (E18) through the end of the fourth postnatal day (P4), then scores were summed over 6 h periods. To generate the graph, 6 h summed scores were plotted for the four cages as box-and-whisker plots. Times shown mark the end of each 6h period scored. Blue shading in each plot shows the “lights out” periods (12 h beginning at 12:00 pm) for each day. All pups were born within a 6-hour period at the beginning of the light phase on the day designated as P0 for that particular cage, as marked with a bar below each graph. Behaviors plotted and colors used are shown above each graph. Blue arrows below each graph show the times of day that samples were taken from similarly co-housed pairs for gene expression and chromatin analysis. **(B)** The frequency with which co-housed virgins groomed pups (plotted in orange) increased significantly over days (*p =* 0.003), as illustrated by a plot focused on 12:00 pm (start of lights out), while the frequency with which virgins groomed mothers (blue) did not change. Values plotted are mean ± standard error. Scoring for these and additional behaviors over the same time periods are summarized in S1 Table.

Consistent with our own observations, a recent study has demonstrated that virgin female mice co-housed with a pregnant dam before and after birth will respond more rapidly and intensely to the pups, with active instruction and encouragement by the mothers [12]. This co-housing paradigm provided us with an excellent opportunity to measure the brain’s functional genomic response to pups in the virgin females over time as they transition to maternal care. To document the timing of this transition, we co-housed four pairs of virgins and pregnant dams and filmed activity in the cages from early pregnancy though the fourth postnatal day. Many aspects of both mothers’ and virgins’ behavior in this paradigm have already been described [12]. However, with the primary goal of establishing a timeline for collection of samples for genomic analysis, we recorded behaviors in a small number (n = 4) cages continuously from just before birth (or embryonic day 18, E18) to postnatal day 4 (P4), and scored behaviors during 5-minute intervals at the top of each hour. We combined scores in each cage over 6-hour periods coordinated with the light/dark cycle, to gather a summary of overall behavioral patterns (S1 Table). For purposes of this study, we were primarily interested in the timing of the shift in pup-directed virgin behaviors. However, to also confirm normal interactions between the two adult females in the cage, we scored the virgins for pup-grooming and also mother-grooming behaviors. Throughout the period, the two females were most often found together, interacting frequently or resting in the shared nest and grooming each other regularly while awake, confirming that the virgin females and mothers were socially engaged. In contrast, although the virgins began to investigate the pups immediately after birth, they did not begin licking and grooming the pups consistently until around P2, after which we increasingly observed the virgins engaged in pup licking/grooming and other related behaviors (Fig 1A; S1 Table). To test the hypothesis that pup-focused grooming increased for the virgins while mother-focused grooming did not, we selected data binned for 6 hours around 12:00 (the beginning of the dark period during lights-out) (Fig 1B). Pup-focused grooming bouts significantly differed across days P1-P4 (repeated measures ANOVA, F_3,9_ = 9.91, p = 0.003), increasing over time, while mother-focused grooming bouts did not significantly differ across days P1-P4 (F_3,9_ = 0.67, p = 0.59). These observations established a time frame for the transition to active pup care.

We confirmed many of the behaviors already described in detail by Carcea and colleagues [12], and those will not be further described here. However, certain behaviors are worth noting. For example, as previously described [12], we observed mothers shepherding virgin cagemates that had wandered off to feed or explore back to the nest; often this involved the mothers grabbing the virgins by the base of the tail and actively pushing them to the nest and pups. Afterward, the mother would herself typically leave the nest to feed, leaving the virgin to care for the pups. Additionally, and not described by Carcea and colleagues, but displayed by all virgins we recorded here, we also observed signs of what we interpreted as an early antagonism toward the pups. Specifically, during the first two postnatal days we observed the virgins grabbing pups in their mouths and actively tossing them or pushing them out of the shared nest (S1 Table). By P3, this behavior was no longer observed, as the virgins spent more time in the nests, licking and grooming the pups with increasing frequency in classic hunched or prone nursing postures (S1 Table), similar to behavior documented for sensitized female rats [17]. Together, these observations suggested that we could indeed capture the transition from the possibly antagonistic, pup-naïve state we observed here, to robust pup affiliation between postnatal days 1 and 3 in this continuous-exposure paradigm.

### Hormone- and neurotransmitter-related genes are dynamically expressed throughout the virgin brain during the first three days of pup interaction

To understand the functional genomic underpinnings of this behavioral transition, we collected RNA from the brains of five virgin females co-housed with a pregnant dam at each of three time points: before birth (2 hr into the dark period of E18) as a control, to compare with females collected at the same time during P1 or P3. At each timepoint, we collected four brain regions from each of 5 females (with the exception of amygdala at E18, for which 4 samples were successfully sequenced), and sequenced RNA for a total of 59 sequenced samples (S2A Table). We collected four brain regions broadly involved in pup response, aversion, affiliation, and reward: hypothalamus, amygdala, striatum, and frontal cortex (see Methods for details of dissections and S1 Fig). We chose to collect RNA from these larger brain regions rather than smaller subregions for two reasons. First, this is the first time that genome-wide data have been collected from alloparenting virgin females of any species; we were not convinced we could accurately predict the correct subregions to sequence at each timepoint. Second, we were interested in tracking the transcriptomic response across the brain over time, and had no clear guidelines for what to expect at later timepoints. The data thus provide a rough snapshot, but one with the potential to generate novel insights into the whole-brain neurogenomic response that can be further investigated in future studies.

**Fig 2:**
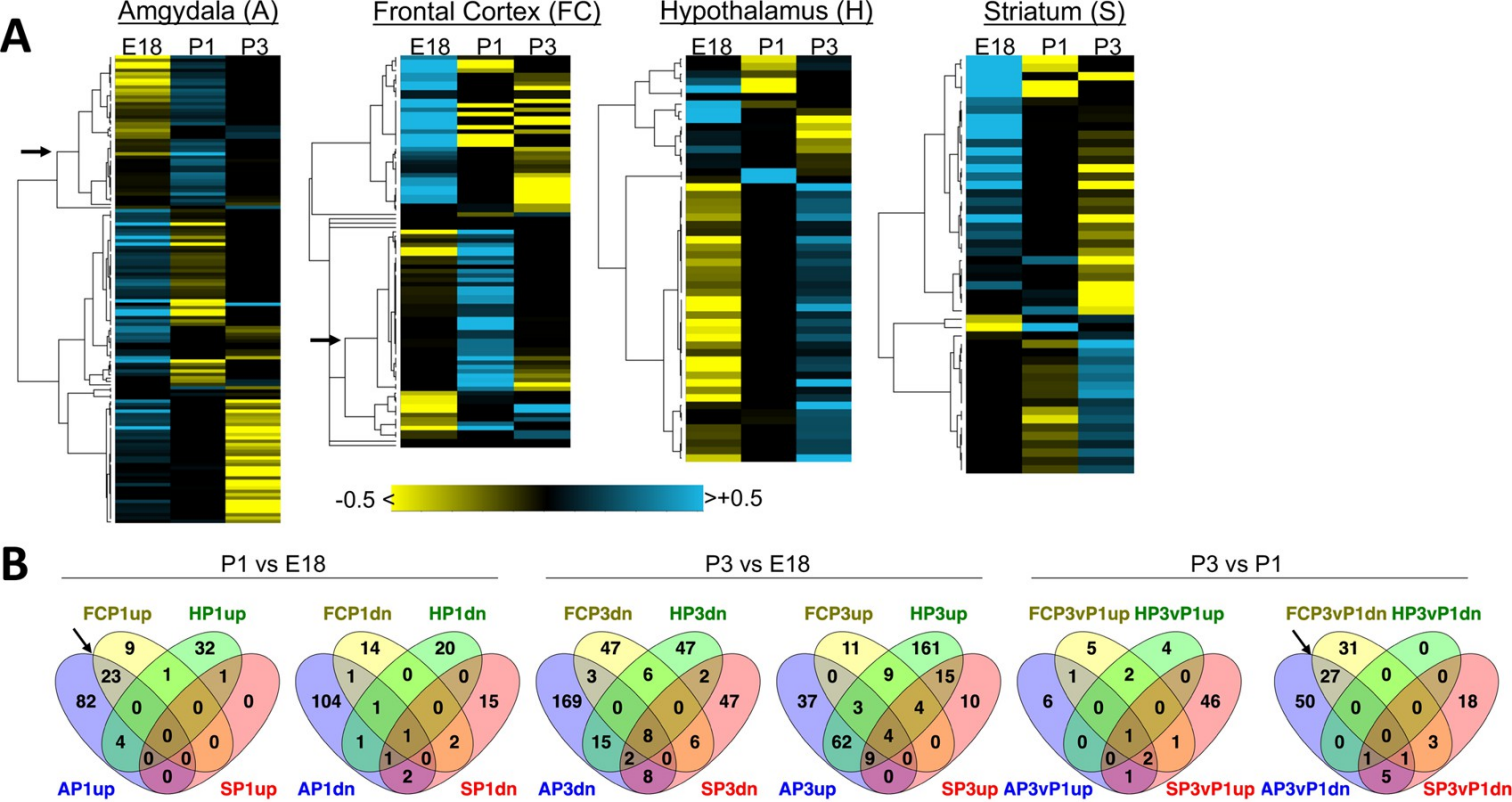
Patterns of transcriptome change across brain regions over time after exposure to pups. **A.** Heatmaps showing patterns of up- (cyan) and down-regulated (yellow) genes in four regions of alloparenting virgins’ brains from E18 to P3, with a focus on genes with fold change >1.5 at any time point within a brain region to highlight the most important patterns. Full gene expression results are detailed in S2 Table. An arrow points to the node in the cluster tree defining groups that overlap significantly between Amydala and Frontal Cortex P1 DEG sets; the same clade is highlighted with an arrow in panel B. **B.** Numbers of genes shared between DEGs (all fdr <0.05, regardless of fold change) from the four brain regions at each time point illustrated as Venn diagrams (generated by Venny 2.1.0, https://bioinfogp.cnb.csic.es/tools/venny/index.html). Statistics for DEG overlap within and between brain regions over time is presented in Table 1. Diagrams showing overlap in up- (up) or down-regulated (dn) genes in each pairwise comparison are shown. A, amygdala; FC, frontal cortex; H, hypothalamus; S, striatum; E18, brains taken from virgin females co-housed with pregnant dams before birth; P1, brains taken during the first postnatal day; P3, brains taken during the third postnatal day.

Each brain region showed a distinct pattern of overlap between differentially expressed genes (DEGs) identified in P1vE18 (labeled P1), P3vE18 (labeled P3), or P3vP1comparisons (Table 1; Fig 2A). For example, in amgydala (labeled A), only 18 genes were shared between the 109 P1 and 115 P3 DEGs (16.8% and 15.6%, respectively); a full 38% of the P1-upregulated genes were down-regulated in P3vP1 comparisons indicating a shift back to baseline levels during the P1-to-P3 transition (this gene cluster marked with an arrow in the amygdala and frontal cortex panels of Fig 2A and 2B), with a hypergeometric *p* value of P1-up/P3vP1-dn overlaps of 1.6E-81 (Table 1). In contrast, half (50.4%) of the genes that were down-regulated at P1 remained down-regulated relative to E18 at the P3 time point (*p =* 6.4E-95, Table 1). The frontal cortex transcriptomic response followed a pattern similar to that of amygdala whereas, hypothalamus and striatum showed relatively small numbers of DEGs at P1, but more robust transcriptomic response at the P3 time point (Fig 2A).

**Table 1:** Significant overlaps between DEG sets.

*Overlaps within brain regions*		*Overlaps between brain regions*	
DEG set 1/DEG set 2	overlap *p* value	DEG set 1/DEG set 2	overlap *p* value
AP1vE18-down/AP3vE18-down	6.4E-95	AP1vE18-down/SP3vE18-down	4.9E-13
AP1vE18-down/AP3vP1-up	8.5E-08	AP1vE18-down/SP1vE18-down	2.0E-08
AP1vE18-up/AP3vP1-down	1.8E-81	AP1vE18-down/HP1vE18-down	4.1E-08
AP1vE18-up/AP3vE18-up	9.9E-26	AP1vE18-up/FCP3vP1-down	7.8E-65
AP3vE18-down/AP1vE18-down	6.4E-95	AP1vE18-up/FCP1vE18-up	1.6E-51
AP3vE18-down/AP3vP1-down	6.9E-51	AP1vE18-up/SP3vE18-up	1.0E-15
AP3vE18-up/AP1vE18-up	9.9E-26	AP3vE18-down/HP3vE18-down	3.1E-35
AP3vE18-up/AP3vP1-up	9.4E-08	AP3vE18-down/SP3vP1-down	2.6E-26
FCP1vE18-down/FCP3vE18-down	1.2E-43	AP3vE18-down/SP3vE18-down	2.3E-24
FCP1vE18-down/FCP3vP1-up	7.5E-11	AP3vE18-up/HP3vE18-up	1.0E-139
FCP1vE18-up/FCP3vP1-down	3.8E-65	AP3vE18-up/HP1vE18-up	6.1E-49
FCP3vE18-down/FCP1vE18-down	1.2E-43	AP3vE18-up/SP3vE18-up	1.1E-25
FCP3vE18-down/FCP3vP1-down	4.9E-18	AP3vP1-down/FCP3vP1-down	1.1E-58
FCP3vE18-up/FCP3vP1-up	1.1E-12	AP3vP1-down/FCP1vE18-up	8.5E-49
SP1vE18-down/SP3vE18-down	2.6E-35	AP3vP1-down/SP3vP1-down	4.7E-14
SP1vE18-down/SP3vP1-up	5.7E-08	AP3vP1-up/FCP3vP1-up	2.6E-15
SP3vE18-down/SP3vP1-down	5.9E-34	AP3vP1-up/SP3vP1-up	7.6E-12
SP3vE18-up/SP3vP1-up	2.5E-48	FCP1vE18-down/SP1vE18-down	9.0E-10
HP1vE18-down/HP3vE18-down	3.6E-30	FCP1vE18-down/SP3vE18-down	1.3E-09
HP1vE18-up/HP3vE18-up	2.2E-68	FCP1vE18-up/HP1vE18-down	1.9E-08
		FCP3vE18-up/HP3vE18-up	2.4E-35
		FCP3vE18-up/SP3vE18-up	5.4E-19
		FCP3vE18-up/SP3vP1-up	7.0E-16
		SP3vE18-up/HP3vE18-up	1.6E-61
		SP3vP1-down/HP3vE18-down	2.4E-18
		SP3vP1-up/HP3vE18-up	1.8E-52

Each DEG set was compared against each other to identify significant overlaps and assign *p* values using a hypergeometric test. Numbers of genes in each overlap are shown in Figs 2B; only overlaps of at least 3 genes were considered. Where more than 3 overlapping DEG sets with *p* < 0.05 were identified for each set the top three are listed; otherwise all non-redundant, significant overlaps are shown. A full list is included in S2 Table.

Considering overlaps between DEG sets in different brain regions, we also found highly significant overlaps between various DEG sets (Table 1). For example, amygdala P1 down-regulated genes overlapped with genes down-regulated in striatum at P1 and P3, and with genes down-regulated in hypothalamus at P1. Amygdala P1 up-regulated DEGs overlapped with high significance with up-regulated genes in frontal cortex at the same time point (*p* = 1.6E-51) and with the genes that were subsequently down-regulated in frontal cortex in the transition to P3 (7.8E-65). On the other hand, genes up-regulated in amygdala at P3 overlapped with highest significance to P3 up-regulated genes in hypothalamus (*p =* 1.0E-139), although most of the overlapping genes were increased or decreased at only modest levels in hypothalamus. Nevertheless, these overlaps suggested coordination between the different brain regions over time.

**Fig 3:**
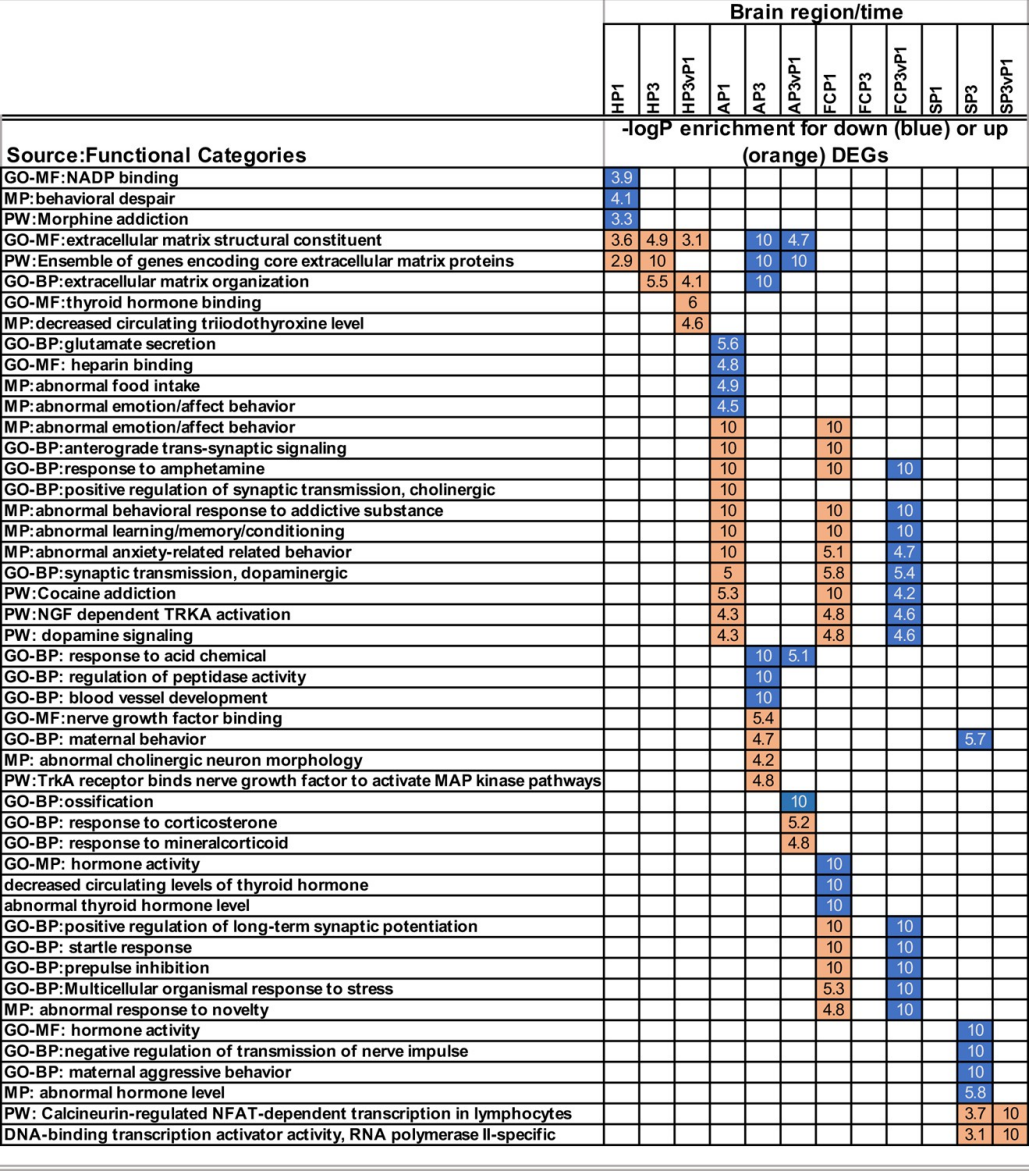
Enrichment of differentially expressed genes in functional categories. Differentially expressed genes (identified at fdr ≤ 0.05 and with absolute value of fold change ≥1.5 in P1 vs E18, P3 Vs E18, and P3 vP1 comparisons, as described in Methods) were used to identify enriched functional categories using the ToppCluster tool (Kaimal et al., 2010), as described in Methods. Categories shown are a representative subset of the full report with all categories, p-values, and DEGs within enriched categories included in S3 Table. All reported categories were enriched with BH corrected p-value of ≤ 0.05; up- or down-regulation and category enrichment levels displayed as a heat map for simplicity. Colored cells denote up- (orange) or down-regulated (blue) Functional Categories in each brain region/time, with numbers denoting the calculated -log p values of enrichment. White squares denote that the category was not enriched in that DEG set.

Focusing first on the hypothalamus in detail, genes encoding neuropeptide hormones oxytocin (*Oxt*) and prolactin (*Prl*) were first up-regulated at P3, when the virgins were beginning to consistently display maternal behaviors (S2B Table); these hormones are central to initiation of maternal response in both mothers and alloparenting virgins [12, 18, 19]. At P1, dopaminergic (DA) signaling components including *Drd1* were down-regulated along with functional categories such as morphine addiction and behavioral despair (Fig 3). However, *Drd1* returned to pre-exposure levels in hypothalamus at P3, at the same time that other genes related to the activity of DA neurons were significantly up-regulated.

Therefore, increased dopamine-related gene expression was simultaneous with the increase of *Oxt* and *Prl* expression in the hypothalamus. This pattern was similar to that observed in mothers at the time of birth and is consistent with the role of DA signaling in OXT and PRL release [20, 21]. It was also consistent with recent observations from single-cell sequencing that show DA neurons in the hypothalamic preoptic area to be activated in maternally behaving animals [22]. The data suggested that a shift to a neuropeptide and neurotransmitter environment favoring stable maternal behavior was developing in the hypothalamus at P3, concomitant with increased expression of alloparenting behaviors in the virgin mice. Furthermore, in light of the hypothesized role of histone modifications on maternal behavior [16, 23, 24], it is worth noting the low-level but coordinated up-regulation of genes encoding chromatin remodeling and binding proteins (*Hdac10*, *Hdac7*, *Sirt6*, and *Sirt7*, *Kmt5c*, *Smarcd3*, *Atrx*, *L3mbtl1*) which we observed in the virgin hypothalamus at P3. This coordinated shift suggested the existence of a subtle but significant epigenetic response–possibly one limited to a small number of neurons, for example, in the MPOA of the hypothalamus, around or perhaps before that time (S2B Table).

In striking contrast to hypothalamus, components of DA signaling including receptors *Drd1*, *Drd2*, *Drd5*, and monoamine transporter *Slc18a2*, were coordinately *up-regulated* in the amygdala at P1, along with genes encoding endogenous opioids, proenkephalin (*Penk*), and prodynorphin (*Pdyn)*. The combined up-regulation of these genes led to P1 enrichment of multiple functional categories indicating that the virgin females were experiencing stress and anxiety during the first day after the birth of the pups (Fig 3; S3 Table). At P3, many of the anxiety-related amygdala DEGs had returned to pre-exposure levels (S2B Table), suggesting that the initial P1 surge of transcription for these genes might be actively silenced. At the same time that the surge of anxiety-related gene expression was suppressed, the GO biological process category “maternal behavior” was identified as being enriched in P3 up-regulated genes (Fig 3).

Frontal cortex tracked the amygdala closely in terms of enriched functional categories, with a few notable exceptions. In particular, genes related to thyroid hormone activity were uniquely downregulated in the virgin frontal cortex at P3, a finding that is especially interesting given the known role of thyroid hormone in maternal care [25] (Fig 3; S3 Table). Finally, in P3 striatum, down-regulated categories were centered on neuropeptide-related genes including those encoding vasopression receptor (*Avpr1a*) and prolactin receptor (*Prlr*). On the other hand, the gene encoding neuropeptide cholecystokinin (*Cck*), which positively regulates striatal dopamine signaling in *Drd2*-expressing neurons [26, 27] was up-regulated in striatum at P3 compared to P1. This event is notable, since *Cck* plays a critical role in the postnatal maintenance of maternal behaviors [28] and mediates responses to anxiety and reward [29, 30]. Together these data indicated that a transcriptomic signature consistent with the maternal response–as it is classically defined by neuropeptide and neurotransmitter gene expression–was beginning to emerge in the virgin mice beginning around P3. In particular, the P1 burst of anxiety-related genes was down-regulated to pre-exposure levels in amygdala and frontal cortex by this time. In contrast with this response in amygdala, DA signaling was *down-regulated* at P1, then *up-regulated* at P3 in the hypothalamus of the pup-exposed virgins, concordant with the onset of maternal behaviors in those mice.

### Comparison to published datasets

#### Parallels to gene expression in brains of new mothers

As referenced above, the differential expression of several key markers and pathways that have been identified in new mothers was also observed in the alloparenting virgins at the P3 time point. An obvious next question was whether and how gene expression aligned more globally between maternal and alloparenting virgin brains. Most published maternal datasets were generated with distinct hypotheses and biological questions in mind, investigating brain regions and time points very different from ours, complicating direct comparisons. Nevertheless, two published data sets warrant some discussion.

In the first series, Gammie and colleagues used microarrays to compare gene expression between virgins (not exposed to pups) and nursing females at P7, after maternal behaviors have been robustly established [31–34]. The same group later completed a meta-analysis of their data to identify genes that were commonly dysregulated across the maternal brain. Despite the differences in methods, time points selected, and brain regions examined, we noted that DEGs identified in the meta-analysis were enriched in similar GO categories, pathways, and disease associations to those we identified as most significantly enriched in the pup-exposed virgin brains: neuron development, addiction, mental health disorders, and pathways involving oxytocin, vasopressin, prolactin, and opioids [35]. The similarity suggests commonalities between maternal behavior and alloparenting behavior.

A second published data series examined maternal gene expression over a wide range of time points pre-and post-partum including P1 and P3, and importantly, used experimental and statistical methodology very similar to ours [36]. However, cortex (neocortex in the maternal study, which includes frontal cortex and additional cortical regions) and hypothalamus were the only brain regions examined commonly in both studies. This similarity allowed us to use formal statistical techniques to measure the degree of overlap between gene sets from our study and this previously collected dataset. Using a hypergeometric test to compare gene expression in pup-exposed virgins and mothers (S2D and S2E Table), we found that DEGs up-regulated in the hypothalamus of P3 virgins correlated positively and most significantly with genes up-regulated in the maternal hypothalamus at P10 (Table 2).

**Table 2:** Significant correlations between differential gene expression in specific brain regions of pup-exposed virgins and new mothers, or virgins and socially challenged male mice.

**Virgin dataset/Maternal Dataset** ^**1**^	***p* value**	**example genes**
FCP3-up/NCP1-down	1.59E-25	*Arc*, *Fos*, *Npas4*, *Celsr3*, *Igsf9b*, *Robo3*
FCP3-up/NCP3-down	1.06E-21	*Arc*, *Fos*, *Npas4*, *Celsr3*, *Igsf9b*, *Robo3*
FCP1-up/NCP1-up	9.32E-15	*Gpr88*, *Pde10a*, *Ppp1r1b*, *Tac1*, *Rasd2*
FCP3-down/NCP10-down	2.54E-11	*Sgk1*, *Nnat*, *Calb2*, *Igsf1*
HP3-up/HP10-up	1.21E-16	*Prl*, *En1*, *Slc6a3*, *Slc10a4*, *Cryab*, *Mif*, *Mfge8*
HP3-up/HP10-down	9.12E-10	*Egr1*, *Fos*, *Junb*, *Lamb2*, *Col6a1*, *Col6a2*
HP3-up/HP3-up	2.16E-07	*Prl*, *Nxph4*
HP3-up/HP1-up	2.99E-07	*Prl*, *Nxph4*
HP1-up/HP10-down	1.23E-06	*Magel2*, *Nr1d1*, *Slc13a4*, *Ogn*
**Virgin dataset/Social challenge dataset** ^**2**^	***p* value**	**example genes**
AP1-up/A120-up	3.67E-54	*Drd1*, *Drd2*, *Rarb*, *Grp88*, *Ppp1r1b*, *Tac1*, *Tcf7l2*
FCP1-up/FC60-up	1.33E-48	*Drd1*, *Drd2*, *Gpr88*, *Ppp1r1b*, *Rxrg*, *Tac1*, *Penk*
AP3-down/A120-up	4.16E-36	*Cdh1*, *Ogn*, *Ccn2*, *Igf2*, *Fmod*, *Sgk1*, *Grin2b*
AP1-down/A120-up	7.16E-35	*Avp*, *Ccn2*, *Grin2b*, *Gucy1a2*
FCP3-down/FC120-up	1.65E-21	*Igsf1*, *Calb2*, *Nnat*, *Trh*, *Gabrq*
AP3-down/A120-dn	3.09E-16	*Slc17a7*, *Tbr1*, *Nrn1*, *Sv2b*, *Lmo3*, *Tafa1*

Hypothalamus (H), Amygdala (A), Frontal cortex (FC) and Neocortex (NC). Highest correlations for all comparisons involving at least 3 overlapping genes are shown, for a full list of comparisons see S2 Table.

The overlapping genes included several involved in DA neuron development and function (*En1*, *Slc6a3* and *Slc10a4*), and neuroprotection and neuroinflammatory processes (*Cryab*, *Mif*, and *Mfge8*). On the other hand, *up-regulated* hypothalamic DEGs from P3 virgins also overlapped with genes that were *down-regulate*d in the maternal hypothalamus at P10 (Table 2); immediate early genes (IEGs) (*Egr1*, *Fos*, and *Junb)* dominated this list along with genes encoding extracellular matrix (ECM) proteins.

We further identified both positively and negatively correlated overlaps in comparisons between virgin frontal cortex and maternal neocortex. DEGs *up-regulated* in virgin frontal cortex at P3 overlapped significantly with DEGs *down-regulated* in neocortex of mothers at P1 and P3 (Table 2); as in hypothalamus, this group of oppositely regulated genes was dominated by IEGs (*Npas4*, *Arc)* and genes involved in ECM, and more particularly ECM proteins involved in axon pathfinding (*Celsr3*, *Igsf9b*, *Robo3*). Why these genes should differ so significantly between virgins and mothers is not clear; however, we speculate that the difference could possibly relate to pathways pre-activated in mothers by the hormones of pregnancy, but not similarly primed in the virgin animals.

Interestingly, there was also significant overlap between DEGs *up-regulated* in both virgin P1 frontal cortex and P1 maternal neocortex. This cluster included genes related to the anxiety-related response that, as noted above, were also up-regulated in the virgin P1 amygdala (*Adora2a*, *Gpr88*, *Pde10a*, *Rasd2*, *Ppp1r1b*, *Tac1*, *Syndigl1*) (Fig 3; S2D, S2E Table); this finding suggested the possibility that mothers might also experience a similar anxiety-related reaction soon after pups were born. More generally, DEGs across the maternal brain showed enrichment in many of the same functional categories detected in brains of the alloparenting virgin mice [36]. Although direct comparison of the same brain regions at similar time points will be required for further clarification, the data are consistent with the idea that virgin and maternal brains activated many of the same pathways in response to pups.

#### Gene expression in P1 virgins closely parallels that of socially challenged males

The similarities between gene expression in mothers and the P3 virgins fit well with the fact that the virgins were beginning to exhibit maternal behaviors around this time. However, the molecular events in the virgin frontal cortex and amygdala around P1 remained something of a puzzle. We noted some similarities between DEGs in the virgin P1 amygdala and frontal cortex and DEGs previously identified in the same brain regions taken from male mice undergoing a territory threat [37], and a hypergeometric test confirmed a robust correlation (Table 2; S2F Table). DEGs up-regulated in P1 virgin amygdala–and particularly those down-regulated in P3vP1 comparisons–showed especially high levels of overlap with genes up-regulated in the amygdala of the socially challenged males; frontal cortex DEGs followed a similar pattern. The overlapping amygdala genes included those associated with dopamine and cholinergic signaling (e.g. *Drd1*, *Drd2*) as well as a large cohort of TF-encoding genes (e.g. *Rarb*, *Foxp1*, *Neurod2*, *Tcf7l2*) (S2G Table). The common up-regulation of these genes in the two social contexts suggested an important and common role. The data were consistent with the interpretation that at P1, the virgin females are experiencing emotions related to fear and threat, marked by a genomic response that was remarkably similar to that operating in the brains of males involved in territory defense. Notably, this threat-related P1 transcriptomic response was largely extinguished in the virgins at P3, as the females began to display maternal behavior toward the pups.

### DEGs cluster into network modules, suggesting regulatory factors with coordinated roles

**Fig 4:**
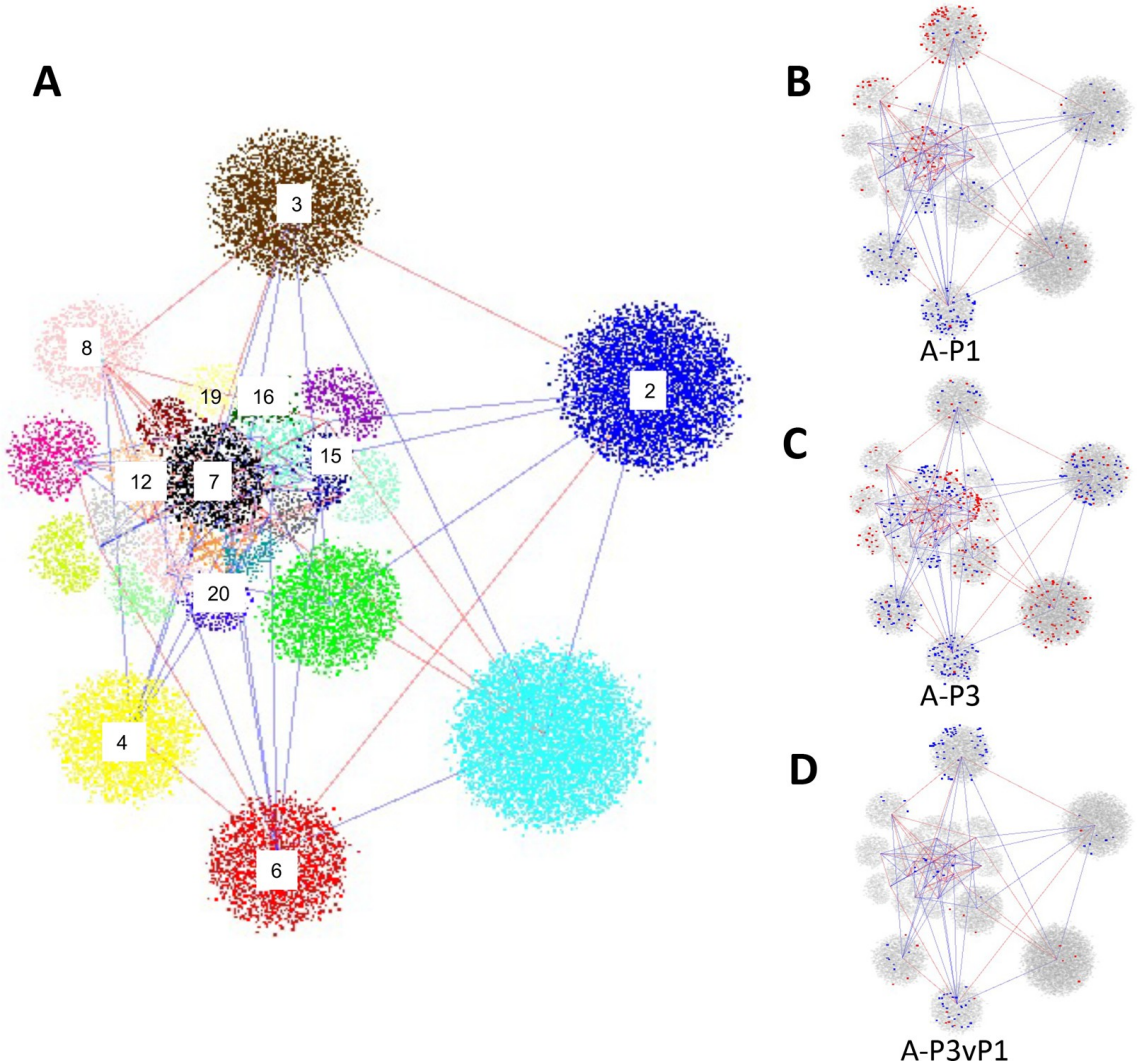
Weighted gene correlation network representing gene expression in four brain regions of virgin female mice. (A) The network, composed of genes expressed in Amygdala, Frontal Cortex, Hypothalamus, and Striatum of pup-exposed and non-exposed virgin controls, consists of 25 modules, each represented by clusters of different color and joined by lines representing positive (blue) or negative (red) eigengene correlations (in both cases showing only those correlations ≥0.6). Numbers have been added to label modules with particular enrichments in amygdala (A) DEGs as referred to in the text. (B-D) representations of the same network but showing the module membership of up- (blue dots) or down-regulated (red dots) DEGs identified in P1 v E18 (A-P1, B), P3 v E18 (A-P3, C), or P3vP1 (A-P3vP1, D) transcriptomic comparisons. Full details of network structure, membership and correlations are provided in S4 Table, with hypergeometric enrichments of genes within modules summarized in Tables 2 and S4E.

To gain insights into the coordination and interactions of regulatory factors involved in these brain transcriptomic events, we used a weighted gene correlation network analysis (WGCNA) approach [38] to generate a co-expression network, including 25 co-expression modules connected by positive or negative edges (Fig 4, full details of network structure in S4A–S4D Table). DEGs from each brain region and time point clustered distinctly within certain network modules, indicating the coordinated regulation of functionally inter-related genes (Tables 3 and S4E). In particular, the threat-related genes that were up-regulated in the virgin amygdala at P1, as well as those up-regulated at P1 and then down-regulated at P3 compared to P1, were especially highly enriched in module 3 (hypergeometric test, *p =* 6.3E-45 and p = 1.3E-22 respectively), with modules 7 and 8 showing a similar but less robust enrichment pattern (Table 3). These three modules together included all but one (98%) of the 54 genes that were similarly up-regulated in P1 virgins and socially challenged males (S2D and S4A Tables). The three positively correlated modules also included several sets of known interacting genes and DEGs with related functions. For example, Module 3 includes *Drd1* and *Penk* together with TF genes *Rarb* and *Foxp1***,** both of which are important to development and activity of development of DA neurons [39, 40]. Module 7 includes *Drd5* and TF-encoding DEG *Tcf7l2*, which has been implicated in fear learning [41]; Module 8 includes *Drd2*, *Pdyn*, *Tac1*, and *Rxrg*, the latter encoding RARB dimerization partner, RXRG. Therefore, the DEGs cluster into modules with inter-related functions, including TFs with known regulatory interactions.

**Table 3:** Significant correlations between differential gene expression and differentially acetylated H3K27Ac peaks in amygdala with specific network modules.

Amygdala DEGs/WGCNA module	*p* value	Amygala TSS-DAPs/WGCNA module	*p* value
AP1-up/module 3	6.3E-45	E18_TSS-DAPs/module 3	4.9E-18
AP1-up/module 7	2.2E-13	E18_TSS-DAPs/module 6	1.6E-12
AP1-up/module 8	1.3E-04	E18_TSS-DAPs/module 2	5.6E-08
AP1-down/module 6	4.0E-33	E18_TSS-DAPs/module 4	7.2E-08
AP1-down/module 4	3.0E-11	E18_TSS-DAPs/module 20	2.0E-03
AP1-down/module 20	4.8E-07	E18_TSS-DAPs/module 8	1.0E-02
AP3-up/module 15	1.8E-23	P3_TSS-DAPs/module 8	4.5E-07
AP3-up/module 1	1.8E-06	P3_TSS-DAPs/module 7	4.2E-06
AP3-up/module 25	8.9E-05	P3_TSS-DAPs/module 24	1.0E-05
AP3-down/module 6	2.2E-29	P3_TSS-DAPs/module 15	3.3E-02
AP3-down/module 19	8.6E-10		
AP3-down/module 16	3.8E-08		
AP3vP1-up/module 6	4.6E-06		
AP3vP1-up/module 6	1.7E-02		
AP3vP1-down/module 3	1.3E-22		
AP3vP1-down/module 6	5.9E-12		
AP3vP1-down/module 7	8.0E-03		

Three top-scoring enrichments with hypergeometric p<0.05, of amygdala DEGs or all top-scoring TSS-DAP-associated genes into specific WGCNA modules, are listed for each DEG or DAP set. For a full list of comparisons of DEGs and DAPs in all brain regions to modules see S4E and S4F Table. AP1, DEGs at P1 compared to E18; AP3, DEGs at P3 compared to E18; AP3vP1, DEGs at P3 compared to P1. TSS-DAPs, DAPs located within 5 kb of a TSS of module-associated genes. E18_TSS-DAPs, peaks enriched in H3K27Ac at E18 compared to P3; P3_TSS-DAPs, peaks enriched at P3 compared to E18.

Other DEG classes clustered into distinct network modules. For example, genes down-regulated at both P1 and P3 clustered together, especially in Modules 4 and 6 (Table 3, S4E Table); P3 up-regulated genes clustered with especially high concentration in Module 15, including the heat shock factor regulator, *Hsf1*, a neuroprotective factor involved in adaptation to stressful experience [42]. Other modules displaying more modest levels of amygdala DEG enrichment reflect brain expression patterns that are strongly correlated with, or anticorrelated to, Modules 3, 6 or 15 and might thus also include regulatory factors involved in cross-module gene activation or repressive effects.

To identify TFs most central to the pup response, we used GENIE3 [43] to reconstruct a gene regulatory network (GRN) with these same data (S5A Table). We then identified TFs in the network with target gene sets that were most highly enriched in DEGs from each brain region and time point (S5B–S5E Table). The data pointed clearly to Module 3 TF *Rarb* as the most central TF in the amygdala P1 transcriptomic response, whereas Module 6 TF genes *Foxc2*, *Osr1*, and *Prdm6*, all three of which are down-regulated in P3 amygdala, dominated the amygdala P3 transcriptomic response (S5B Table); brain functions of these Module 6 TFs are not known. Module 15 TF gene *Hsf1*, which is itself up-regulated in hypothalamus at P3, was the most highly associated with DEGs in that brain region and time point (S5C Table). Of potential interest, *Snapc4*, a module 15 TF that activates expression of small nuclear RNAs [44, 45] figured prominently in hypothalamus at both time points, suggesting a role for regulation of RNA splicing in the hypothalamic response.

### Dynamic changes in amygdala chromatin at the P3 time point

The behavioral adaptations that follow maternal and alloparenting experiences have long been thought to involve epigenetic factors [20, 23]. We therefore expected that histone modifications could play a key role in the virgins’ transition to maternal care. In particular, we hypothesized that key genes involved in the threat reaction we observed at P1 might be actively silenced by these mechanisms around the time that the virgins began to display maternal behaviors at P3. We tested this hypothesis by carrying out chromatin immunoprecipitation (ChIP) in chromatin from each of the four brain regions from virgin females co-housed with mothers at E18 and P3. For these ChIP experiments, we used an antibody specific to histone 3 acetylated at lysine 27 (H3K27Ac), a marker for active promoters and enhancers [46].

**Fig 5:**
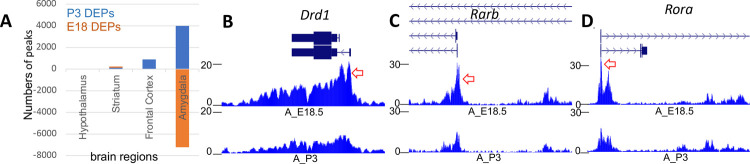
Dramatic changes in the amygdala chromatin landscape accompanies the transition to maternal-like behaviors in alloparenting virgin mice. (A) Relative numbers of TSS-associated differentially enriched peaks (DAPs), as measured by >2-fold change in detected levels of H3K27Ac, in the four brain regions tested in this study. Positive numbers represent peaks more enriched at P3 than E18 (P3 DAPs); negative numbers represent less enriched (E18 DAPs) peaks. (B-E) Examples of DAPs in *Drd1*, *Rarb1* (both up at P1 and then down-regulated at P3), *Rora* (down-regulated at P1 and P3 compared to E18), showing normalized H3K27Ac profiles in amygdala chromatin at E18 (A_E18, top track) and P3 (A_P3, bottom track) for each gene. Red arrows in each panel point to examples of significant differentially acetylated peaks. Full ChIP profiles are available online as a UCSC Browser track hub at https://trackhub.pnri.org/stubbs/ucsc/public/allo.html), and data are available in S5 and S6 Tables.

Consistent with our previous results [37], the ChIP profiles revealed tens of thousands of acetylated chromatin peaks in every brain region for both pup-exposed and non-exposed females (S6 Table). Peaks were positively correlated with gene expression, as expected. Since differentially acetylated peaks (DAPs) offer a window into chromatin dynamics, we identified peaks with highest potential to impact gene expression by focusing on DAPs in which the relative levels of H3K27Ac were at least two-fold higher or lower in brains collected at P3 compared to E18 consistently in biological replicate samples at fdr < 0.05 (S7 Table). Surprisingly although peaks were identified in similar numbers overall in the each of the four brain regions, DAPs were virtually absent in the chromatin samples from hypothalamus at P3 and were found in relatively low numbers in frontal cortex and striatum at this time point as well. In striking contrast, chromatin from the P3 amygdala contained thousands of DAPs, either increased in H3K27Ac enrichment (5325 DAPs) or decreased (7209 DAPs) at P3 compared to E18 (Fig 5A, S7A Table). Many of these differential peaks could mark distal enhancers, but with the goal to maximize the chances of linking DAPs to specific DEGs, we focused our attention on the smaller subset of DAPs located within 5 kb of the TSS of an annotated gene (TSS-DAPs). Altogether we found 2738 TSS-DAPs enriched at H3K27Ac at E18 compared to P3 (hereafter referred to as E18 DAPs), and 1040 TSS-DAPs enriched in H3K27Ac at P3 compared to E18 (P3 DAPs).

Interestingly, the genes associated with E18 or P3 TSS-DAPs, respectively, clustered into network modules that were also enriched for up- or down-regulated DEGs (Tables 3 and S4F). For example, amygdala E18 DAPs were particularly enriched for linkage genes in modules 3 and 6 (hypergeometric *p =* 4.87E-18, *p =* 1.64E-12, respectively), whereas P3 DAPs were most likely to be associated with genes in Modules 7 and 8 (*p =* 4.52E-7, *p* = 4.24E-6, respectively). The TSS-DAPs were associated with 138 amygdala DEGs, including 22 TSS containing P3 DAPs, 115 TSS containing E18 DAPs, and 1 TSS hosting DAPs of both types (S6B Table). Given that amygdala DEGs were up-and down-regulated in roughly equal numbers (Fig 2) the preponderance of down-regulated genes in TSS-DAPs suggested that alterations in the chromatin landscape at P3 were primarily focused on silencing amygdala genes. The DEGs associated with E18 DAPs were enriched specifically and significantly in network module 3 (hypergeometric *p* = 4.9E-18), suggesting an especially important role for histone de-acetylation in silencing this coregulated cluster of threat-associated genes. Examples include amygdala E18 DAPs associated with TSS of Module 3 genes, *Drd1* and *Rarb* (Fig 5B and 5C) and associated with the primary alternative promoter of *Rora* (Module 6 TF gene down-regulated at both P1 and P3) (Fig 5D). Together the data suggested that activities of many key genes involved in pup response are associated with differential enrichment of the H3K27Ac mark, particularly in the amygdala.

### Enrichment of binding motifs points to mechanistic insights

To obtain further information regarding the potential activity of TFs in the pup response, we searched for enrichment of known TF binding motifs (TFBMs) in the 2X P3 and E18 amygdala TSS DAPs. The search identified REST/NRSF binding motifs as the top enrichment within P3 TSS-DAPs (E18 enriched compared to P3); although *Rest* itself was not identified as differentially expressed, the data suggest that chromatin around REST TFBMs was being rendered less accessible between E18 and P3 in amygdala of the pup-exposed virgins. This finding is of interest, because REST is a central regulator of neuron differentiation and plasticity [47], and also plays a role in stress resilience in adult brain [48]. The search also identified enrichments for motifs in the E18 DAPs that are recognized by TFs encoded by amygdala DEGs, including P3 down-regulated genes *Mef2c* and *Rora*. Consistent with their expression, the TSS of both genes were associated with E18 DAPs (S7B Table). Together these data indicate that histone deacetylation events evident at P3 serve to not only reduce levels of *Mef2c* and *Rora* gene expression, but simultaneously, to reduce the accessibility of both TFs to their target genes. Notably, both TF genes have been associated with deficits in social behavior [49–51] and *Rora* has been implicated in maternal behavior specifically [52], supporting a functional role. Notably, *Mef2c* target genes predicted by GRN analysis were significantly and specifically enriched in pup-driven DEGs in amygdala; MEF2C target genes were also predicted to include a notably high number of other TFs (S5B Table). These data suggest that MEF2C may play a role as one of the central hubs coordinating the amygdala transcriptomic response.

Some notable TFBM enrichments were also detected in P3 DAPs. For example, we noted enrichment of FOX family TFs including the specific TFBM of the protein encoded by DEG, *Foxp1* which was up-regulated in amygdala at P1, then down-regulated between P1 and P3 (S7 Table). However, this FOX motif could potentially also be recognized by other family members including FOXC2; *Foxc2* was down-regulated in the P3 amygdala and was predicted in the GRN to be central to the P3 response (S5 Table). Because we did not measure chromatin at P1, the initial timing of these epigenetic events is not discernible. However, the data suggest that while TSS-linked targets of REST, RORA and MEF2C became less accessible, targets of FOX family proteins became more accessible via epigenetic modifications during the postnatal period.

## Discussion

With the goal of understanding molecular mechanisms that underlie the transition to maternal behaviors, we investigated the behavioral, transcriptomic, and epigenomic response of virgin females as they were co-housed with mothers and newborn pups over a period of several days. A recent study used this same paradigm to show that alloparenting virgins are instructed in pup care by co-housed mothers [12], and we observed a very similar pattern of behaviors in the mothers and virgins we tested here. Along the way, although virgin female mice did not display an obvious aversion toward the pups, we also observed evidence of an initial antagonism during the first two postnatal days; these antagonistic behaviors gave way to increasing levels of attention to the pups, with the virgins increasingly licking, grooming and huddling over the pups by postnatal day 3.

The data presented here revealed a dramatic and dynamic neurogenomic shift that coincided with successful maternal instruction and the activation of oxytocin neurons in the virgin brain. In particular, at P1 we observed a transcriptomic signal of fear and anxiety in the virgin hypothalamus, frontal cortex and especially in the amygdala, in the form of a gene expression pattern that correlated with high significance to that observed in territory-challenged males [37]. Despite the lack of obvious aversion, these data provide support for the notion that indeed–at least within this co-housed paradigm–virgin female mice did initially perceive the pups as anxiety-inducing, or even possibly threatening. If the threat signal was related to the aversive response observed in rats and other species, the data would be consistent with the hypothesis that pup aversion and defensive behaviors share a common brain circuitry [4], and would suggest a shared molecular mechanism for diverse types of threat response as well. Several TF genes implicated by gene expression, network co-expression, chromatin analysis, and/or motif-enrichment analysis were similarly up- or down-regulated in the pup-exposed virgins and socially challenged males, suggesting crucial roles for these TFs in regulating this shared molecular signature of social threat.

Published data support the roles of several of these TFs in threat/anxiety response. For example, the RARB:RXRG dimer’s activities in amygdala have been linked to expression of anxiety-related phenotypes [53], and *RORA* is associated with enhanced fear response in humans [54] and mice [55]. Furthermore, *Rora* mutant mouse mothers do not retrieve, care for, or suckle their young [56]; the data presented here support further investigation of this gene’s role in maternal amygdala. Furthermore, other TF genes implicated in the shared threat signature are associated with social-behavior phenotypes, including *Tcf7l2*, which was up-regulated in the amygdala of virgins at P1 as well as in socially challenged males [37] and is important for fear-learning and adaptation [41]. Our data suggest that these TFs have coordinated roles in the fear response.

We hypothesize that these and other networked TFs work together to modulate the response to pups, and the networks developed from our dataset suggests a robust framework of positive and negative gene interactions that coordinate this behavioral switch over time. The expression of the threat-related TF genes was extinguished along with the pulse of dopamine signaling after the rise of oxytocin, prolactin, and other neuropeptides by P3, paving the way for a shift in the virgin females’ behavior toward the pups; we surmise that this shift was driven, at least in part, by a substantial level of histone modification in the amygdala. Many of the genes that returned to normal expression levels between P1 and P3 in amygdala were associated with chromatin that was differentially enriched for H3K27Ac, consistent with an active epigenetic silencing in that brain region at P3.

Histone modifications have been implicated in the development of maternal behaviors in both mothers and alloparenting virgins, although most published studies have focused on the hypothalamic MPOA as the primary site of this epigenetic response [16, 23, 57]. These studies have shown that *suppression* of HDAC activity–and thus *inhibition* of chromatin silencing–in hypothalamus is key to driving the females’ maternal response. Surprisingly therefore, we found no evidence of changes in H3K27Ac modification in the P3 hypothalamus. However, a hypothalamic signal could have been missed for several reasons. First, histone modifications in a small subregion, like the MPOA, could have been diluted in analysis of the whole dissected hypothalamus. Second, a sweep of histone modification might have taken place at a different time after pup exposure. However, the systemic application of HDAC inhibitors used in published studies could impact acetylation across entire brain; the massive histone shift we did observe in amygdala was weighted toward *de-acetylation*, or chromatin closure, along with the silencing of associated differentially expressed genes. These findings could suggest that HDAC activity plays a *positive* role in the acquisition of maternal behavior, although examination of earlier time points or in different or more specifically dissected brain regions (the MPOA, for example) might have provided a different view. These data give us guidelines for when and where to focus in future studies. However, they also suggest an important role for amydgala that has not been previously explored. For example, although deacetylation in amygdala may be critical in quenching the threat response once established, *suppression* of deacetylation in hypothalamus (and/or other brain regions) before P1 might have prevented the establishment of the aversive/fear response in the first place. Possibly relevant to this hypothesis is the coordinated up-regulation of histone deacetylase and chromatin remodeling genes we detected in the hypothalamus at P3; this signal could reflect the trace of earlier epigenetic events associated with the expression of fear and anxiety in the virgins before they transitioned to active pup care.

This is the first study to investigate global genomic and transcriptomic events in the context of alloparental care. Our studies highlight a special role for the amygdala in the switch to alloparenting behavior in this context, a hypothesis that is consistent with the known functions of amygdala in maternal behavior and bonding [58, 59]. As underscored by human brain imaging studies, maternal behavior involves a global brain response that unfolds over an extended period of pre- and postnatal time [60]; in the pup-exposed virgins, we detected just the start of this behavioral transition during the third postnatal day. Nevertheless, the mechanisms involved in this transition to intensive pup care could be relevant to a successful transition to motherhood as well. Although it is not yet possible to determine whether a similar response is activated, or actively suppressed, at some time around birth in the maternal amygdala, this question is an important one in the context of maternal bonding and infant care. Especially given the similar up-regulation of anxiety-related genes we identified in published data from the maternal neocortex, we speculate that a similar active suppression of a threat/anxiety program may occur in the amygdala of new mothers, and that dysregulation of this program could underlie the failure of mother-infant bonding, post-partum anxiety and depression. Addressing this hypothesis will offer a novel perspective on the causes of these very common, painful and highly consequential human maladies.

## Materials and methods

### Mice and behavioral analysis

All work with mice was done under the approval of the IACUC at the University of Illinois, Urbana Champaign. Mice were housed in a temperature-controlled room in a reverse 12h/ 12h light-dark cycle. Six-week-old female mice (C57BL/6J x C3HJ F1 hybrids, with an agouti coat to allow clear distinction with the black-coated virgins) purchased from the Jackson Laboratory were impregnated, and co-housed with age-matched virgin female C57BL/6J mice during pregnancy and through the early post-partum pregnancy. To record behavior, four pairs were filmed in clear-topped cages continuously using a Samsung SCB-2000 CCTV Camera with iSpy 64 v7.2.1.0 CCTV software. Behavior was scored in each cage (0 or 1) in 5-minute snapshots at the top of each hour from the day before and until the end of the fourth day after birth, with scores combined over 6 h periods for each cage to generate the illustrative plot in Fig 1. To test the hypothesis that pup-grooming behaviors increased over time, while mother-grooming behaviors did not, we performed one-way repeated measures ANOVA in R (v4.0.4) using the rstatix package (v0.7.0). The anova_test() function in rstatix automatically assesses repeated measures data for the assumption of sphericity. Scores for these and other behaviors are also presented in S1 Table. The 5-minute video snapshots are provided as S1 Video with video clips of specific and unusual behaviors noted in the text provided as S1 File; additional video is available on request.

### Dissections and RNA preparation

Dissections were performed as described in detail previously [37] with the addition of the striatum, which is illustrated in S1 Fig. Briefly, mice were euthanized by cervical dislocation followed by rapid decapitation. Their brains were removed and sectioned in a coronal slicing mouse brain matrix. A total of three cuts were made: two cuts separated by 4 mm defined by the rostral and caudal aspects of the hypothalamus and a third cut bisecting these two cuts. The hypothalamus, frontal cortex, striatum, and amygdala were dissected from the resultant brain slices. Upon completion of these dissections, focal brain regions were placed into cryotubes, snap-frozen in liquid N2, and stored at -80°C until downstream processing. Samples were prepared for sequencing from the four dissected brain regions of five mice per condition (E18, P1, P3). RNA isolation and QC were completed as described previously, with libraries prepared robotically at the Roy J. Carver Biotechnology Center at University of Illinois, also as described in [37].

### Gene expression analysis

Illumina sequencing libraries were generated with the TruSeq Stranded mRNA HT kit (Illumina) using an Eppindorf ePMotion 5075 robot and were sequenced to a depth 45–60 million reads per sample on Hi-Seq 2500 instruments at the Roy J. Carver Biotechnology Center at the University of Illinois. All sequencing data generated in this study have been deposited to the GEO database under Accession number GSE184549. Pairwise comparisons of E18, P1 and P3 samples were completed as previously described in detail [37], with results provided in S2 Table. For functional analysis, genes that were found to be differentially expressed at fdr < 0.05 were first filtered for absolute fold change >1.5, and uploaded to the ToppCluster web analysis tool [61] using default conditions (Bonferroni correction, fdr <0.05). Selected categories are summarized in Fig 2, with full ToppCluster Results reported in S3 Table.

### Network analysis

We used signed WGCNA (Langfelder & Horvath, 2008) to generate networks from the data from all individuals, brain regions, and time points, as described in depth in our previous study [37]. Eigengenes calculated for each module were used to generate module correlations; details of module structure, module gene content, eigengene correlations, and hypergeometric enrichments are presented in S4 Table. After log-transforming our data using voom+limma, we filtered zero variance genes, selected a soft thresholding coefficient of 3, then used a signed Pearson correlation analysis with a minimum module size of 30. Images in Fig 3 were generated using version 3.7.1 of Cytoscape [62].

To reconstruct the GRN, we obtained a list of 1523 potential transcription factors in mouse from Animal Transcription Factor Database [63]. GENIE3 [43] was applied on the expression data of 37991 genes in 53 conditions consisting of various brain regions (H = hypothalamus, A = amygdala, FC = frontal cortex, and S = striatum) and different time points (E18, P1-P3) to score the relative significance of each TF-gene interaction. (Auto-regulatory relationships were excluded). To construct a GRN, for each gene we collected up to top five TF regulators of that gene as predicted by GENIE3, additionally requiring that the TF-gene pair have a Spearman’s correlation of at least 0.5 (in absolute value) and a GENIE3 score of at least 0.005. The resulting GRN included 92717 interactions involving 1400 unique TFs and 21156 genes (S5A Table). To assess the significance of TF regulons in different brain regions, for each TF we computed the enrichment of its regulon (gene set predicted to be regulated by the TF) for DEGs from each brain region, using hypergeometric test (S5B–S5E Table).

### ChIP tissue preparation, chromatin immunoprecipitation, and library preparation

ChIP was performed essentially as described in detail in our previous study [37]. Briefly, brain tissue dissected from 3 animals was pooled, homogenized, and fixed in PBS with 1% formaldehyde for 10 minutes. Nuclei were prepared from the fixed cells and stored at -80°C until use. Thawed nuclei were sonicated using a BiorupterTM UCD-200 (Diagenode, Liège, Belgium) sonicator, and fragmented chromatin was processed for ChIP with 2 ug histone H3K27Ac antibody per sample (Abcam ab4729), using one million nuclei for each IP. IPs were performed in biological replicate, with one pool of 3 samples in each replicate, as previously described. Libraries were prepared from eluted DNA using KAPA LTP library kits (KK8230) using Bioo Scientific index adapters, size-selected using AmpureXP beads (Beckman Coulter, Brea, CA, USA) and quality checked by Qubit 2.0 and Bioanalyzer (Agilent 2100). Samples were sequenced to a depth of 20-30M reads per replicate on an Illumina HiSeq 2500 sequencer using a TruSeq SBS sequencing kit, version 4, in single-end format with fragment length of 100 bp. Base calling and demultiplexing into FASTQ files was done using bcl2fastq v1.8.4 software (Illumina, San Diego, CA, USA).

### ChIP-Seq bioinformatics

ChIP sequencing reads were mapped with Bowtie2 [64] to the UCSC Mus musculus mm9 or mm10 genome, using default settings and analyzed for peaks using HOMER (Hypergeometric Optimization of Motif EnRichment) v4.7 [65], as previously described [37]. Differential chromatin peaks were identified in biological replicates using the HOMER getDifferentialPeaksReplicates.pl script, looking for any peaks that changed at least two-fold between conditions with an FDR cutoff of 0.05. Known motif discovery was performed with the HOMER findMotifsGenome.pl script using default settings with 201bp peak regions extracted from all histone peaks or only differential histone peaks. Chromatin profiles are available online as a UCSC Genome Browser track hub at https://trackhub.pnri.org/stubbs/ucsc/public/allo.txt).

## Supporting information

S1 Fig Diagram of the mouse brain showing details of brain region dissections used in this study. (PDF)

S1 Table Behavior scoring: Behaviors (scored 0 or 1 for 5 minutes at top of each hour) summed over 6 hr periods in cages 1–4 ending at times shown. (XLSX)

S2 Table RNA-seq gene expression analysis and full results of DEG hypergeometric tests. (XLSX)

S3 Table Full ToppCluster gene ontology results for each DEG set. (XLSX)

S4 Table Complete results of WGCNA analysis including hypergeometric tests for module enrichments. (XLSX)

S5 Table Full results of GENIE3 GRN analysis and lists of TFs with scores for enrichment for DEGs from each brain region within target gene lists. (XLSX)

S6 Table **A.** H3K27Ac peaks detected by HOMER as significant in duplicate ChIP samples for amygdala. **B.** H3K27Ac peaks detected by HOMER as significant in duplicate ChIP samples for frontal cortex. **C.** H3K27Ac peaks detected by HOMER as significant in duplicate ChIP samples for hypothalamus. **D.** H3K27Ac peaks detected by HOMER as significant in duplicate ChIP samples for striatum.
(ZIP)

S7 Table H3K27Ac peaks detected as Differentially Acetylated Peaks (DAPs) in comparisons between virgin brain regions at the E18 and P3 time points. (XLSX)

S8 Table Transcription factor binding motifs enriched within DAPs in each brain region. (XLSX)

S1 Video Videotaped examples of unusual virgin behaviors noted in the text. (MP4)

S1 File (MP4)
