# Supplementary figures and images for: An epigenomic shift in amygdala marks the transition to maternal behaviors in alloparenting virgin female mice
Source: PLoS One. 2022 Feb 22;17(2):e0263632. doi: 10.1371/journal.pone.0263632 (PMC8863255; doi:10.1371/journal.pone.0263632)

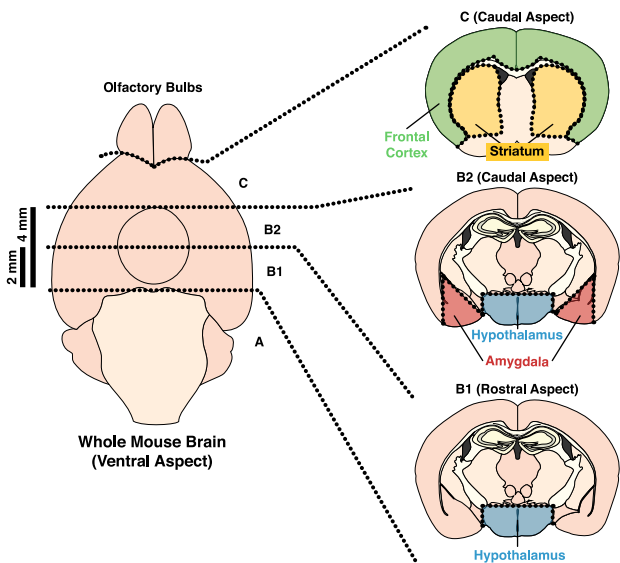

Supplement: S1 Fig — (PDF) [file pone.0263632.s001.pdf]
